# Mismatch negativity in older adults and its relationship with the cognitive and behavioral aspects of central auditory processing

**DOI:** 10.6061/clinics/2021/e1830

**Published:** 2021-02-01

**Authors:** Mirtes Brückmann, Karina Carlesso Pagliarin, Michele Vargas Garcia

**Affiliations:** I Programa de Pos-Graduacao em Disturbios da Comunicacao Humana, Universidade Federal de Santa Maria (UFSM), Santa Maria, RS, BR; II Departamento de Fonoaudiologia, Universidade Federal de Santa Maria (UFSM), Santa Maria, RS, BR

**Keywords:** Auditory Evoked Potentials, Auditory Cortex, Hearing Loss, Aged, Cognition, Hearing Tests

## Abstract

**OBJECTIVES:**

This study aimed to describe and compare the performance of older adults with normal hearing and hearing impairments in mismatch negativity (MMN), correlate MMN with cognitive tasks and central auditory processing (CAP), and identify normal values for MMN in older adults.

**METHODS:**

This study had 54 participants. The Montreal Cognitive Assessment (MoCA) was used to assess cognition and the random gap detection test (RGDT), dichotic digit test (DDT), and speech to noise (SN) test were used to evaluate CAP. MMN was elicited with the verbal stimulus /da/ (frequent) and /ta/ (rare), and the latency, amplitude, duration, and area were analyzed.

**RESULTS:**

When comparing the normal-hearing group to those with hearing loss, there was no significant difference in MMN. When correlating MMN with MoCA, RGDT, DDT, and the SN test, there was a weak correlation between the MMN amplitude and the RGDT and DDT. When comparing the MMN of participants with normal and altered cognitive aspects and those with normal and altered DDT, the MMN duration was found to be affected by the DDT. The mean latency value of the MMN in the normal-hearing group was 199.8 ms, the amplitude was -2.2 µV, area was 116.1 µV/ms, and duration was 81.2 ms.

**CONCLUSION:**

Mild hearing loss did not influence MMN. There was no correlation between MMN and cognitive aspects, and there were weak correlations with CAP. Alterations in CAP led to longer durations in MMN. Normal values for MMN in adults aged between 60 and 77 years were generated.

## INTRODUCTION

Considering the rapid aging of the global population, geriatric research has become increasingly necessary, as there has never been such a large number of people at such an advanced age (1). Among the main sensory alterations that occur as a result of aging is presbycusis (2), which can cause different losses in different individuals, such as central auditory processing disorder (CAPD).

Audiologists are already aware of the importance of using hearing tests that evaluate both peripheral and central hearing and the benefit of this union for a more accurate diagnosis (3). Undoubtedly, it is one of the methods that contributes most to the rehabilitation of older adults.

CAPD can occur because of presbycusis, but also by the process of natural aging. During aging, there is a decrease in the number of neurons responsible for synapses that adequately transmit sound stimuli (2). It is normal for such alterations to occur from the age of 60 years in the temporal regions responsible for hearing; they further progress to the neocortex, which is responsible for cognitive performance (4).

The evaluation of the central portion is performed using behavioral tests that evaluate central auditory processing (CAP) and electrophysiological properties, such as long latency evoked auditory potentials (LLEAPs). LLEAPs are advantageous because, in addition to hearing assessment, it allows one to assess attention and memory (5,6). However, few studies have used LLEAP mismatch negativity (MMN) in older adults. In addition, electrophysiological tests allow for non-verbal answers during the procedure, and they differ from behavioral tests that can suffer from interference from external, environmental, and physical conditions. It is important to emphasize that in MMN, the capture of auditory potentials occurs automatically, without the need for a participant to pay attention to the sound stimulus. This allows for different possibilities in analysis regarding aspects of attention in this population (5).

From this perspective, tests that evaluate cognition can work in tandem to guide the rehabilitation process. Cognitive decline can occur continuously and regularly for intensive processing tasks such as processing speed, working memory, and long-term memory (7,8).

Therefore, the justification for this study is centered on expanding assistance to older adults, providing a more accurate diagnosis and, consequently, greater possibilities for rehabilitation. It is also intended to establish the relationship between MMN and behavioral and cognitive tests in older adults.

Thus, this study aimed to describe and compare MMN in older adults with normal hearing and those that are hearing-impaired, correlate MMN with cognitive and CAP tasks, and identify normal values for MMN in older adults.

## METHODS

This was a cross-sectional, descriptive, and quantitative study approved by the local Research Ethics Committee on Human Beings (#3326307). All individuals invited to participate in the research were instructed about their free and spontaneous participation and signed a Free and Informed Consent Form, which contained all the procedures to be performed.

Older adults of both sexes from social group participants in the region and those who sought audiological evaluation from a public speech therapy service were invited. Individuals aged 60 years or older were considered older adults (9).

The eligibility criteria were as follows: age ≥60 years; auditory thresholds within the normal range in both ears (10), or mild sensorineural hearing loss (up to 40 dBHL) in the average frequencies from 500 to 4000 Hz (10) in both ears; with a maximum difference of 10 dB in the quadri-tonal mean between the ears; be literate; no history of using an individual sound amplifier; no external or middle ear alterations; no visual difficulties according to self-reported data that could impede the performance of the tasks; no diagnosis of neurological or psychiatric disorders reported by participants or that were evident.

After collecting the eligibility data, the behavioral tests of the dichotic digit test (DDT), random gap detection test (RGDT), and speech in noise (SN) test were performed inside an acoustically treated booth, with the aid of a two-channel audiometer (FA-12 Fonix Hearing Evaluator, Frye Electronics; Beaverton, OR) and earphones (TDH-39P, Telephonics; Farmingdale, NY). The test was transmitted by a computer that remained attached to the audiometer.

The DDT was performed to evaluate the auditory ability of figure-background for verbal sounds (11). Two digits were presented in each ear simultaneously, performed in the stage of binaural integration, at an intensity of 50 dB above the average of the frequencies of 500, 1000, and 2000 Hz, using the Brazilian version by Pereira and Schochat (11). The participants were instructed to repeat the four digits presented in both ears, regardless of the order. We considered normality values as correct answers of 78% or more for normal-hearing older adults and correct answers ≥60% or more for those with hearing loss (11).

The SN test was performed to assess auditory closure using the Brazilian version by Pereira and Schochat (11). We presented 25 monosyllabic words in each ear, with an intensity of 40 dB above the average of the frequencies of 500, 1000, and 2000 Hz, with the presence of white noise in an ipsilateral way, in a signal/noise ratio of +10 dB. The participants were instructed to ignore the noise and repeat the words that they understood. Normal values were considered as correct answers of 70% or more, both for those with normal hearing thresholds and for those with hearing loss (11). This normality criterion is suggested in the literature for individuals with normal hearing thresholds. However, because there is no standard for individuals with hearing loss, it was decided to use the same value as the normal-hearing sample, considering that the sample had only mild hearing loss.

The RGDT (12) was performed to assess the auditory ability of temporal resolution. Pure tones were presented at frequencies of 500, 1000, 2000, and 4000 Hz, with time intervals between the tones ranging from 0 to 40 ms in random order. The test was presented in binaural mode at 50 dB above the average of the frequencies of 500, 1000, and 2000 Hz. In cases where the participant did not detect the gaps in 40 ms, the expanded version of the test was applied, in which the intervals between the tones varied from 50 to 300 ms. In this test, participants were asked if they heard one or two whistles. We considered the shortest time interval after which the participant started to consistently identify the presence of two tones at all frequencies. From this, a mean of the value between the four frequencies was calculated. As a criterion of normality, values of up to 51 ms were established for those with normal thresholds and up to 79 ms for those with hearing loss (13).

The tests were selected to perform auditory processing screening. Therefore, the most relevant skills for older adults were considered. In addition, an evaluation that was neither extensive nor tiresome was recommended, as it could otherwise interfere with performance. Thus, the purpose of this CAP evaluation was to capture alterations present in older adults with complaints about the ability to perform figure-background and auditory closure when they are exposed to unfavorable environments. Temporal resolution is also considered a skill affected by age, which compromises speech understanding; the RGDT is considered the most sensitive test to detect alterations in this ability (14).

In addition, the DDT and RGDT can be applied to older adults with hearing loss. The DDT, for example, has normality criteria for older adults with sensorineural hearing loss (11). The RGDT has already been applied in older adults with such characteristics (14,15), and recently, a study demonstrated reference values for older adults with hearing loss (13). The SN test was chosen even though it does not have normality values for older adults with hearing loss, due to the importance of assessing the ability of auditory closure, which in most cases is related to hearing complaints.

After the CAP tests, participants were evaluated using the Montreal Cognitive Assessment (MoCA, version 8.1) (16), which assesses eight cognitive domains: visual-spatial/executive ability, naming, attention, language, abstraction, late evocation/memory, and orientation. As a normality criterion, there are values above 24 points for a total of 30 (17). For people with educational levels equal to or below 12 years, one point is added to the result.

Finally, all participants underwent MMN testing using two-channel Smart-EP equipment (Intelligent Hearing Systems, Miami, FL). To capture the potentials, silver electrodes were fixed at specific points according to the International Electrode System 10-20 as follows: active electrode in Fz, ground electrode in Fpz, and reference electrodes in the right and left mastoids, with the aid of MaxxiFix^®^ (Neurovirtual) electrolytic paste and micropores. Before placing the electrodes, the skin was cleaned with Nuprep^®^ (Weaver and Company) exfoliating gel for better conduction of the electrical signal.

The participants remained awake, sitting in a comfortable armchair, and the potentials were captured and visualized on a computer to which the equipment was attached.

MMN was analyzed with the verbal stimulus of da/ta, a synthetic and unnatural speech stimulus, in which /da/ represents a frequent stimulus and /ta/ a rare stimulus (18), presented in a traditional Oddball paradigm, in which the rare stimulus was emitted at random among several frequent stimuli (19). The duration of the stimulus /da/ was 206.2 ms and 220.3 ms for /ta/. The impedance was maintained at a level equal to or less than 3 kΩ.

Auditory stimuli were transmitted via earphones in a binaural way and were guaranteed from 20 to 25 dBNS in all participants (20,21). They were instructed to watch a subtitled film without audio on a computer, and asked to remain as quiet as possible, pay attention only to the film, and ignore the sound stimulus (19).

The stimuli were presented at a speed of 1.9 stimuli/s, with a rare stimulus probability of 20%. There were 750 stimuli presented in total, in an attempt to obtain at least 150 rare stimuli (19,22,23). The tracing was filtered using a 1.0-Hz low-pass filter and a 30.0-Hz high-pass filter. The registration window used was 50 ms before stimulation and 512 ms after stimulation (24). Up to 10% of artifacts from the total stimulus were allowed.

Wave analysis was performed by the researchers and two expert judges in the area to achieve a consensus. The presence or absence of MMN was analyzed, and if present, the variables analyzed were latency, amplitude, duration of the potential, and valley area.

**Figure 1:**
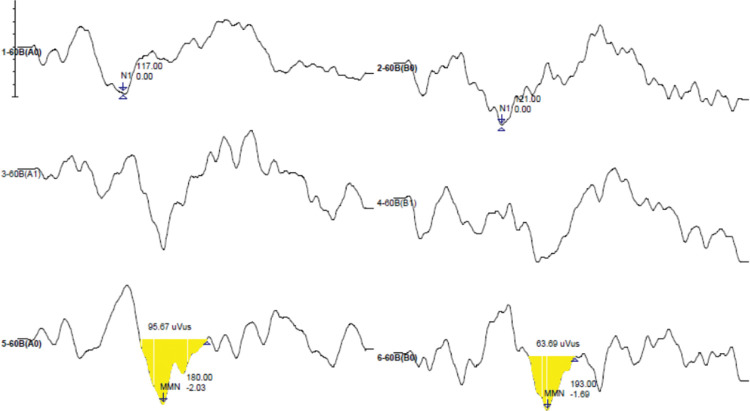
Example of mismatch negativity latency, amplitude, area, and duration. Examination data obtained from a patient evaluated in this study.

MMN was considered as a negative peak, obtained by the difference in curves (resulting wave) by subtracting the response curves to the frequent stimulus from the response curves to the rare stimulus, visualized in a latency after N1 (25,26) and measured in milliseconds. To mark the amplitude, the pre-stimulation line was used as the zero point for the “size” of the valley as the maximum measure, considered as the most negative point where the latency was marked to the zero point, not including the positive part of the wave. In cases where the line had not reached zero, the marking ended earlier (18). A minimum amplitude of 0.3 µV was considered as suggested by the equipment manual. When the amplitude marking was created, the valley area was automatically registered, measured in microvolts per milliseconds (µV/ms). Duration (ms) was measured as the difference between the initial and final latencies of the potential. Figure 1 shows a marked MMN model, in which it is possible to observe the latency, amplitude, area, and duration.

For the sample calculation (27), an analysis was performed based on older adults who presented normality by the DDT. Standard deviations were obtained for MMN latency. A significance level of 5%, power of 80%, and sampling error of 50 ms were considered. Considering these values, the optimal sample size for a representative sample was 21 participants.

After collecting the data, all results were recorded in Microsoft Excel (Microsoft, Redmond, WA) and later analyzed using Statistica 9.0 software (Dell, Round Rock, TX). The Shapiro-Wilk test was used to assess normality, in which variables with *p*≥0.05 were considered normal and those with *p*≤0.05 were non-normal.

After this analysis, the majority of variables were non-normal. Therefore, non-parametric tests were used for further analysis. The Mann-Whitney U test was used for comparisons, and Spearman's correlation coefficient was used for correlations. Significant results were considered when *p*≤0.05, with a 95% confidence interval.

## RESULTS

Initially, 85 older adults participated, but only 54 had the necessary criteria for the study. Table 1 describes the general data of this sample for sex, age, education, and hearing characteristics.

There were 33 older adults with normal hearing with a mean age of 65.8 years (SD=3.9) and 21 with hearing loss with a mean age of 68.4 years (SD=5.3). There was no significant difference in the mean ages of participants with or without hearing loss (*p*=0.086). The mean years of formal study in the group with normal hearing was 11.1 years (SD=5.9) and 9.8 years (SD=4.6) in those with hearing loss. There was no significant difference between the groups in terms of years of education (*p*=0.433).

Table 2 presents the descriptive and comparative analysis of the MMN variables (latency, amplitude, area, and duration) in participants with normal hearing thresholds and those with hearing loss. Of the 54 older adults who participated in this study, 47 of them elicited MMN.

As shown in Table 2, hearing loss did not influence MMN in older adults. As such, all participants were analyzed as a single group. Afterwards, a correlation analysis was carried out between the MMN variables and the cognitive (MoCA) and CAP (RGDT, DDT, SN) tests (Table 3).

As shown in Table 3, most correlations were not significant, except for that between amplitude and the RGDT and DDT in the right ear. However, these correlations were weak.

Table 4 shows a comparison between participants with normal scores altered by the MoCA and those with normal scores altered by the DDT, in terms of MMN variables. This analysis was performed to identify whether all participants could be part of the analysis to define criteria for normality in MMN.

As shown in Table 4, only DDT results were able to influence the duration of MMN, as it was higher for those who presented with altered DDT. Although the number of subjects were different in this comparison between normal and altered groups, older adults who presented with altered DDT were excluded to achieve MMN normality values (Table 5).

Thus, of the 47 older adults who had elicited MMN, seven participants who presented alterations in CAP screening through DDT were excluded. An analysis was made for normal MMN values (Table 5) from 40 normal-hearing older adults or those with mild hearing loss who presented normality in the MoCA, considering that these two factors did not influence MMN responses. The average age of participants in this new sample was 67.06 years (SD=4.67) and the average years of education was 11.08 years (SD=5.43).

## DISCUSSION

Hearing is an important aspect to consider during aging, since presbycusis is somewhat predictable with advanced age as well as CAPD (28) and cognitive alterations (7,8). The present study aimed to analyze the performance of older adults with normal hearing and those with hearing loss in MMN and compare this performance with cognitive and CAP aspects, in addition to determining normal values of MMN for older adults.

The results of this study showed that mild sensorineural hearing loss does not interfere with MMN performance. A study that evaluated adults and older adults with hearing loss using Net Amps 300 equipment with the application of a white noise stimulus found that MMN amplitude was significantly reduced and the latency prolonged in those with hearing loss of up to 70 dB in the mean frequencies from 500-4000 Hz (21). Two other studies used NeuroScan, Inc (Herndon, Virginia, USA) equipment and the syllabic set /ba/ and /ga/. One of them evaluated adults with and without hearing loss and found that in mild losses, there was a decrease in amplitude and a slight increase in latency, but in moderate losses there was a significant decrease in amplitude and increase in latency, whereas MMN was not elicited in significant losses (29). The other study investigated children aged 9 and 10 years with mild to moderately severe hearing loss and found no difference in MMN between the groups, but the authors suggest that this may have occurred because the children in the group with hearing loss were hearing-aid users, which may have contributed to the maintenance of hearing discrimination ability (30).

In the current study, the older adults with hearing loss had never used a hearing-aid; therefore, the same conclusion cannot be made as in the aforementioned study. However, older adults have greater sound and linguistic experience due to their age compared to children, which can compensate for some alterations caused by mild hearing loss. However, given the findings of the current study along with what is presented in the literature, it is believed that mild loss does not really have the capacity to cause significant alterations in MMN because the studies that showed alterations in amplitude and latency in this potential were performed in greater losses.

However, such a hypothesis cannot be stated clearly because the studies presented have been carried out in different populations and different age groups with different sound stimuli and equipment, which can also interfere with MMN results. In addition, few studies in the literature have presented the effects of hearing loss in MMN in different age groups, especially in older adults.

When correlating MMN with the MoCA and CAP results (Table 3), a positive correlation was seen between the MMN amplitude and RGDT; that is, as the RGDT value increases (worsens), MMN amplitude also increases (improves), which is not an expected result because alterations in temporal resolution result in difficulties in identifying small acoustic variations. In this case, the MMN amplitude was not negatively affected by the temporal resolution and there was sufficient recruitment of neurons for the amplitude to increase, perhaps due to the differences in sound stimuli used in MMN, as it was not minimal and could be perceived even by individuals with a higher threshold of temporal resolution. However, this correlation was weak as its coefficient value was between 0.3 and 0.5 (31).

The same occurred with the MMN amplitude and the DDT in the right ear, but with a negative correlation; that is, as the percentage of correct answers in the DDT decreased (worsened) in the right ear, the MMN amplitude increased (improved), which is also not expected because it is understood that the poorer the auditory processing, the poorer the discrimination of sounds in the electrophysiological evaluation would be and vice versa. In this case, the correlation was weak and did not occur in the left ear. There was no correlation between the SN test and MMN.

Given the results, it was evident that MMN was not negatively affected by difficulties in behavioral skills assessed by the RGDT, DDT, and SN test. No studies that carried out an analysis of MMN with CAP tests in older adults were found, which made it difficult to compare and discuss the results in this population. The influence of CAP alteration on MMN has already been shown in children (32), but there are studies that have not found such influences (33,34). However, there is a difference in the CAP tests applied as well as in the electrophysiological equipment used, in addition to other characteristics presented by the children. Therefore, it remains an area that needs to be better explored in the different age groups to understand the real influences of CAP tests on MMN.

When comparing normal and altered older adults in terms of cognitive and CAP aspects through screening using MoCA and DDT, it was observed that only MMN duration was affected by the alteration in DDT, becoming longer (Table 4). MMN duration is understudied in the literature, and it is not yet well-defined.

As older adults with altered DDT results show longer MMN durations, this may indicate that they need more time to discriminate the rare stimulus, considering that there is a great similarity between the rare and frequent stimuli, despite the detection time (latency) being similar to older adults with normal DDT results.

However, cognition is unlikely to cause impairment in MMN (Table 4), as they were not correlated (Table 3). The findings of the current study regarding cognitive aspects are contrary to other studies that demonstrate an influence by cognition on MMN, causing losses (35,36). These studies may have showed this result due to a more advanced cognitive decline in the studied populations or due to the diagnoses having been carried out with other methods, which may be more precise regarding cognitive alteration. In the population of the current study, the fact that they are socially active older adults with a maximum of mild hearing loss may guarantee better cognitive conditions (37), and consequently these aspects may not affect MMN.

Thus, to generate normality values for MMN, older adults aged between 60 and 77 years with normal CAP and normal hearing or mild sensorineural hearing loss were considered, regardless of cognitive conditions and an average schooling of 11.08 years. Regarding the normal values found for the MMN variables (Table 5), it was observed that the mean latency is similar to those reported in studies of adults, which include 150 and 250/275 ms (38,39). The average amplitude in the current study was also similar to that of another studies carried out with older Brazilians using other equipment (40).

A limitation of this study is the absence of older adults with higher degrees of hearing loss for comparison. In the future, it is believed that MMN would not as a diagnostic instrument for alterations in CAP or cognition, but as a biomarker that can monitor the effects of therapy or auditory training in older adults.

## CONCLUSION

MMN was observed in normal-hearing older adults with mild sensorineural hearing loss. Mild hearing loss did not influence the latency, amplitude, area, or duration of this potential. When correlating MMN with CAP tests, there were some weak correlations with amplitude, but alterations in CAP did not negatively affect MMN. Individuals with altered DDT had a longer MMN duration. Cognitive aspects do not seem to influence MMN responses.

It was possible to generate normality values for older adults aged between 60 and 77 years with normal hearing or hearing loss. The average latency value was 199.8 ms, the amplitude was -2.2 µV, the area was 116.1 µV/ms, and the potential duration was 81.2 ms.

## AUTHOR CONTRIBUTIONS

Brückmann M was responsible for the data collection, data tabulation, statistical analysis, and manuscript preparation. Pagliarin KC was responsible for the manuscript writing and revision. Garcia MV was responsible for the orientation and revision of the manuscript.

## Figures and Tables

**Table 1:** Sample description regarding sex, age, education, and hearing.

Total participants	n=54
Sex (female/male)	38/16
Mean age (SD)	66.8 (4.64)
Mean Schooling (SD) years	10.59 (5.41)
Hearing (Normal-hearing/Hearing loss)	33/21

SD, standard deviation.

**Table 2:** Descriptive and comparative analysis of mismatch negativity variables for older adults with normal hearing thresholds and with hearing loss.

Variables	Hearing	n (47)	Mean (±SD)	Median	Min-Max	*p*-value^1^
Latency	Normal	28	213.9 (±58.1)	194.8	136.5-326.0	0.205
	Loss	19	192.1 (±56.5)	172.0	144.5-358.5	
Amplitude	Normal	28	-2.1 (±1.1)	-1.8	-0.8-(-5.5)	0.229
	Loss	19	-2.5 (±1.4)	-2.1	-0.4-(-5.9)	
Area	Normal	28	106.8 (±74.4)	83.5	16.7-278.5	0.340
	Loss	19	139.4 (±121.9)	106.6	13.7-565.2	
Duration	Normal	28	83.5 (±29.8)	79.0	37.0-157.5	0.914
	loss	19	85.8 (±31.4)	78.0	50.5-177	

1 Mann-Whitney U Test.

n, number of participants; SD, standard deviation; Min, minimum; Max, maximum.

**Table 3:** Correlation analysis between mismatch negativity variables with MoCA, RGDT, DDT, and SN Test in older adults (n=47).

Variable	r	*p*-value^1^	Variable	r	*p*-value^1^
Latency & MoCA	0.094	0.529	Area & MoCA	-0.043	0.772
Latency & RGDT	0.014	0.925	Area & RGDT	0.244	0.102
Latency & DDT-RE	0.141	0.345	Area & DDT-RE	-0.268	0.068
Latency & DDT-LE	-0.054	0.718	Area & DDT-LE	-0.163	0.275
Latency & SN-RE	-0.083	0.578	Area & SN-RE	-0.154	0.301
Latency & SN-LE	-0.052	0.731	Area & SN-LE	0.014	0.927
Amplitude & MoCA	-0.047	0.752	Duration & MoCA	-0.139	0.351
Amplitude & RGDT	0.339	0.021	Duration & RGDT	0.193	0.199
Amplitude & DDT-RE	-0.301	0.040	Duration & DDT-RE	-0.240	0.104
Amplitude & DDT-LE	-0.164	0.269	Duration & DDT-LE	-0.142	0.341
Amplitude & SN-RE	-0.125	0.403	Duration & SN-RE	-0.214	0.149
Amplitude & SN-LE	0.013	0.933	Duration & SN-LE	-0.027	0.856

1 Spearman Correlation.

RE, right ear; LE, left ear; MoCA, Montreal Cognitive Assessment; RGDT, random gap detection test; DDT, dichotic digits test; SN, speech in noise; r, correlation coefficient.

**Table 4:** Comparison analysis between those with normal and altered MoCA and those with normal and altered DDT for mismatch negativity variables.

Variables	MoCA	n	Mean (±SD)	*p*-value^1^	Variables	DDT	n	Mean (±SD)	*p*-value^1^
LAT	Normal	20	211.8 (±60.7)	0.498	LAT	Normal	40	199.8 (±55.5)	0.091
	Altered	27	200.2 (±56.3)			Altered	7	235.6 (±65.9)	
AMP	Normal	20	-2.2 (±1.6)	0.349	AMP	Normal	40	-2.2 (±1.3)	0.386
	Altered	27	-2.2 (±0.9)			Altered	7	-2.4 (±0.9)	
AREA	Normal	20	122.8 (±128)	0.439	AREA	Normal	40	116.1 (±102.3)	0.169
	Altered	27	117.8 (±67.2)			Altered	7	141.8 (±53.3)	
DUR	Normal	20	81.3 (±30.5)	0.349	DUR	Normal	40	81.2 (±29.3)	0.045*
	Altered	27	86.7 (±30.1)			Altered	7	103.0 (±29.8)	

1 Mann-Whitney U Test.

LAT, latency; AMP, amplitude; DUR, duration; MoCA, Montreal Cognitive Assessment; DDT, Dichotic Digit Test.

**Table 5:** Normative data referring to mismatch negativity variables for normal hearing or mild hearing loss, with normal or altered results in the Montreal Cognitive Assessment (age, 60-77 years).

Variables	n	Mean	Standard Deviation
Latency	40	199.8	55.5
Amplitude	40	-2.2	1.3
Area	40	116.1	102.3
Duration	40	81.2	29.3
